# Reduced Sleep-Like Quiescence in Both Hyperactive and Hypoactive Mutants of the Galphaq Gene *egl-30* during lethargus in *Caenorhabditis elegans*

**DOI:** 10.1371/journal.pone.0075853

**Published:** 2013-09-20

**Authors:** Juliane Schwarz, Henrik Bringmann

**Affiliations:** Max Planck Institute for Biophysical Chemistry, Göttingen, Germany; Brown University/Harvard, United States of America

## Abstract

Sleep-like states are characterized by massively reduced behavioral activity. Little is known about genetic control of sleep-like behavior. It is also not clear how general activity levels during wake-like behavior influence activity levels during sleep-like behavior. Mutations that increase wake-like activity are generally believed to also increase activity during sleep-like behavior and mutations that decrease wake-like activity are believed to have decreased activity during sleep-like behavior. We studied sleep-like behavior during lethargus in larvae of *Caenorhabditis elegans*. We looked through a small set of known mutants with altered activity levels. As expected, mutants with increased activity levels typically showed less sleep-like behavior. Among these hyperactive mutants was a gain-of-function mutant of the conserved heterotrimeric G protein subunit Galphaq gene *egl-30*. We found, however, that an unusual semidominant hypoactive mutant of *egl-30* also had reduced sleep-like behavior. While movement was severely reduced and impaired in the semidominant *egl-30* mutant, sleep-like behavior was severely reduced: the semidominant *egl-30* mutant lacked prolonged periods of complete immobility, reduced spontaneous neural activity less, and reduced responsiveness to stimulation less. *egl-30* is a well-known regulator of behavior. Our results suggest that *egl-30* controls not only general activity levels, but also differences between wake-like and sleep-like behavior.

## Introduction

Sleep is a complex behavioral state that is found in mammals. But also all other animals that have been carefully studied, display quiescence behavior. Several of these quiescence behaviors fulfill behavioral criteria that define sleep in mammals such as an absence of voluntary movement, reversibility, increased arousal threshold, assumption of a specific posture, homeostatic regulation, and changes in the nervous system [1]. If quiescent behavior fulfills these criteria, it is often called a *sleep-like state* or *sleep-like behavior* [2]. It seems like these sleep-like states are much less complex compared with sleep in mammals and it is unclear how sleep and sleep-like states are evolutionarily related. Quiescent behavior can also be found in *C. elegans*. Larvae go through four larval stages called L1 to L4. At the end of each larval stage, animals molt. Before ecdysis, larvae go through a phase of quiescent behavior lasting two hours that is called lethargus [3]. Recent work has shown that quiescence behavior during lethargus fulfills behavioral criteria that define sleep-like behavior such as absence of voluntary movement, reversibility, increased arousal threshold, a specific posture, homeostatic regulation, and changes in the nervous system [2,4-7].

Unlike sleep in mammals, quiescence behavior in *C. elegans* is not controlled by a circadian rhythm, but by the molting cycle. In the presence of food, the molting cycle has a periodicity of 8-10 hours, which is shorter than the circadian cycle. Interestingly, the *period* gene that controls circadian rhythms in other systems has a *C. elegans* homolog called *lin-42*. Like the period gene, *lin-42* mRNA levels oscillate with the wake-sleep-like cycle [8]. Knockout of *lin-42* causes defects in the timing of the molting cycle and the timing of the sleep-like behavior [9]. Thus, circadian rhythm genes control the molting cycle in *C. elegans*.

Several pathways have already been implicated in the control of sleep-like behavior during lethargus. One of the first mutants found was a gain-of-function mutant in *egl-4*, a cyclic nucleotide dependent kinase. *egl-4gf* mutants show ectopic phases of quiescence that are characterized by an absence of locomotion and pharyngeal pumping [2,10]. Ectopic sleep-like quiescence has also been achieved by overexpression of either *lin-3* or *osm-11*, implicating Epidermal Growth Factor signaling and Notch signaling in the control of sleep-like quiescence [11,12]. In addition to the conditions that increase sleep-like behavior, also mutants with decreased sleep-like behavior have been described. *Acy-1* gain-of-function mutants are hyperactive and sleep-like behavior is interrupted by phases of high activity [2,6]. Mutation of components of the EGF pathway, such as let-*23* or *plc-3*, does not abolish quiescence, but quiescence is often punctuated by bursts of activity [11]. Also, loss-of-function mutants of the Notch signaling pathway have reduced quiescence [12].

*egl-30* is a known regulator of neural function and also of many behaviors such as pharyngeal pumping and locomotion [13]. One of its major functions in neurons appears to be facilitation of neurotransmitter release [14]. Both hypomorphic loss-of-function mutations and semidominant mutation in *egl-30* were shown to severely reduce behavioral activity levels [13,15,16].

Here we found that an unusual semidominant mutant in *egl-30* was lacking substantial periods of immobility during lethargus. We characterized sleep-like quiescence behavior during L1 lethargus in the semidominant allele *egl-30*(*n715sd*), and compared it with the gain-of-function allele *egl-30*(*tg26gf*) [17]. We assayed spontaneous movement, neural and muscle calcium activity, and responsiveness to stimulation and found that both a hyperactive and a hypoactive mutant of *egl-30* have reduced sleep-like behavior during L1 lethargus.

## Materials and Methods

### Worm maintenance and strains used

**Figure 1:**
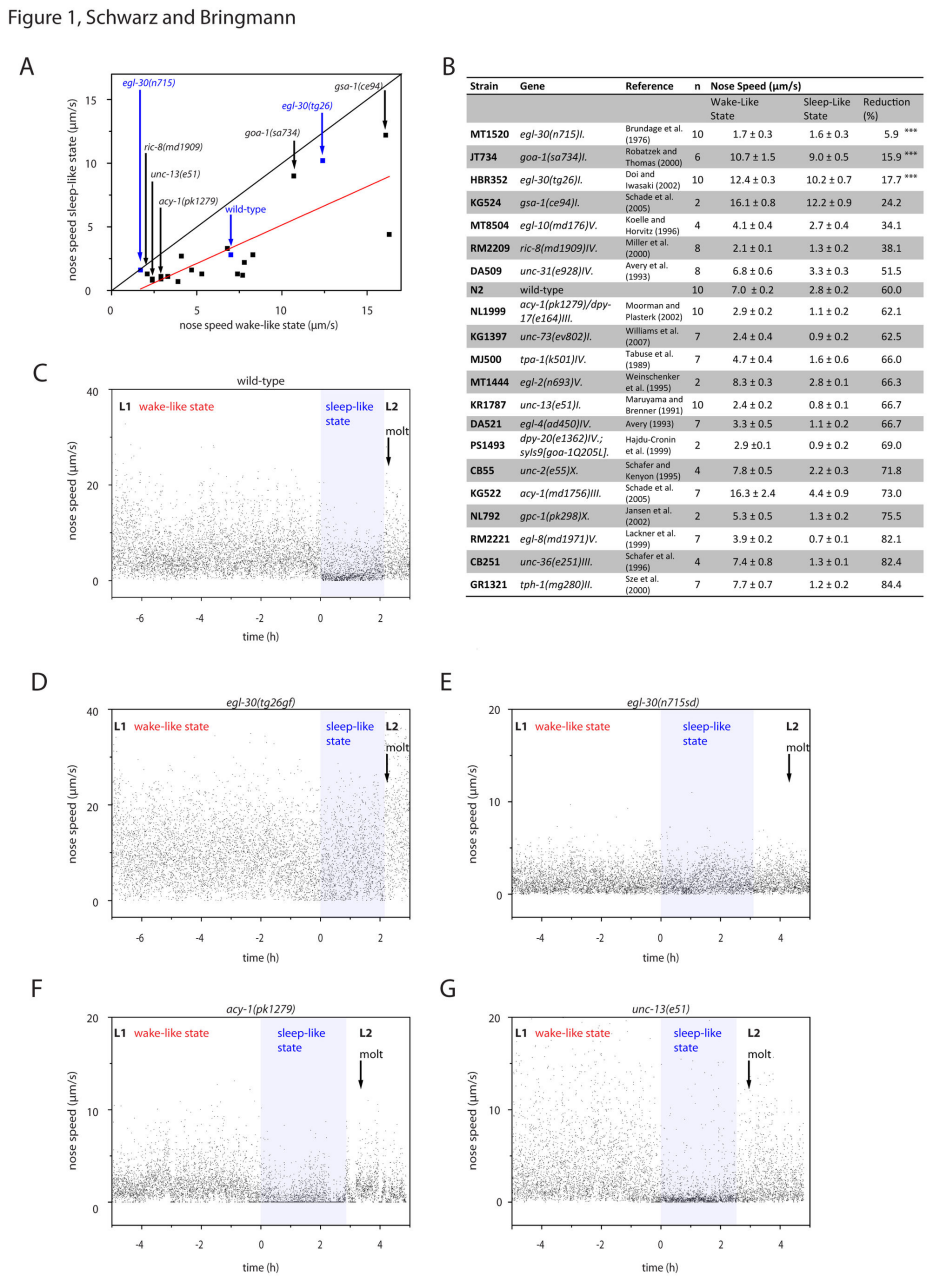
Quiescence in mutants with known alterations of activity levels. **A** Correlation between movement during wake-like and sleep-like states in wild type and in several mutant strains. Each black dot represents one strain. We filmed worms with a frame rate of 0.2/s. The red line represents a linear fit. B Strains assayed for the analysis in A. Values displayed show mean ± SEM. ***denotes statistical significance from wild type (two-sample t-test, p<0.001) **C-G** individual examples of nose speed measurements for one wild type (C), one *egl-30*(tg26gf) (D), one *egl-30*(n715sd) (E), one *acy-1*(pk1279), and one *unc-13*(e51) example worm (G). The sleep-like state as defined by the absence of feeding is shown in light blue. A black arrow indicates the timing of the cuticle shedding (molt). The beginning of lethargus was arbitrarily set to 0h.

*C. elegans* was maintained on NGM plates as described [18]. After bombardment into *unc-119*(*ed 3*), all insertions described below were backcrossed two times against N2 to remove *unc-119*(*ed 3*). The following strains and alleles were used in addition to the strains specified in Figure 1B:

N2: wild type
CG21: *egl-30*(*tg26*) *I*; him-*5*(*e1490*) *V*.
MT1434: *egl-30*(*n686*) *I.*
MT2609: *egl-30*(*n715n1190*) *I.*
DA823: *egl-30*(ad805) *I.*
DA1084: *egl-30*(ad806) *I.*
HBR352: *egl-30*(*tg26*) I. (Created from CG21 by backcrossing 2x into wild type to remove the him background, this strain was used for all *tg26* experiments).
MT1520: *egl-30*(*n715*) *I.*
HBR460: *egl-30*(*n715*) *I*,*goeIs42*[*pegl-30::egl30cDNA-mkate2::egl-30-3*'*utr*,*unc-119*(*+*)]*.*
HBR569: *egl-30*(*n715*) *I*,*goeEx279*[*myo-3::egl30cDNA-mkate2::egl-30-3*'*utr*,*unc-122::rfp*,*unc-119*(*+*)]*.*
HBR602: *egl-30*(*n715*) *I*,*goeIs125*[*pmyo-3::egl30cDNA-mkate2::egl-30-3*'*utr*,*unc-119*(*+*)]*.*
HBR604: *egl-30*(*n715*) *I*,*goeIs126*[*pmyo-3::egl30cDNA-mkate2::egl-30-3*'*utr*,*unc-119*(*+*)]*.*
HBR599: *egl-30*(*n715*) *I*,*goeIs127*[*punc-119::egl30cDNA-mkate2::egl-30-3*'*utr*,*unc-119*(*+*)]*.*
HBR600: *egl-30*(*n715*) *I*,*goeIs128*[*punc-119::egl30cDNA-mkate2::egl-30-3*'*utr*,*unc-119*(*+*)]*.*
HBR4: *goeIs3*[*pmyo-3::gcamp3.35::unc-54-3'utr, unc-119(+)*] *V.*
HBR235: *egl-30*(*tg26*) *I, goeIs3*[*pmyo-3::gcamp3.35::unc-54-3'utr, unc-119(+)*] *V.*
HBR418: *egl-30*(*n715*) *I, goeIs3*[*pmyo-3::gcamp3.35::unc-54-3'utr, unc-119(+)*] *V.*
HBR204: *goeIs24*[*punc-119::sl1::gcamp3.35::sl2::mkate2-unc-54-3’utr, unc-119(+)*]*.*
HBR213: *egl-30*(*n715*) *I, goeIs24*[*punc-119::sl1::gcamp3.35::sl2::mkate2-unc-54-3’utr, unc-119(+)*]*.*
HBR220: *egl-30*(*tg26*) *I, goeIs24*[*punc-119::sl1::gcamp3.35::sl2::mkate2-unc-54-3’utr, unc-119(+)*]*.*
HBR205: *goeIs22*[*pmec-4::sl1::gcamp3.35::sl2::mkate2-unc-54-3’utr, unc-119(+)*]*.*
HBR216: *egl-30*(*tg26*) *I, goeIs22*[*pmec-4::sl1::gcamp3.35::sl2::mkate2-unc-54-3’utr, unc-119(+)*]*.*
HBR206: *egl-30*(*n715*) *I, goeIs22*[*pmec-4::sl1::gcamp3.35::sl2::mkate2-unc-54-3’utr, unc-119(+)*]*.*
AQ2026: *ljIs105*[*sra-6::chr2::yfp, unc-122::gfp*]*.*
HBR6: *egl-30*(*tg26*) *I, ljIs105*[*sra-6::chr2::yfp, unc-122::gfp*]*.*
HBR7: *egl-30*(*n715*) *I, ljIs105*[*sra-6::chr2::yfp, unc-122::gfp*]*.*

### Microcompartment culture

Agarose microcompartment culture was carried out as described [19]. We used 190µm x 190µm compartments. We previously tested whether behavioral parameters change with increasing the microcompartment dimensions and we did not find any differences [19]. We used the slightly smaller version of the microcompartments because they allowed fluorescence imaging [19]. To obtain eggs containing mutants carrying *egl-30*(*n715sd*), gravid adults were placed into a 10µl drop of S-Basal placed on a glass coverslip and were cut open using needles, which released the eggs from the animals. The egg suspension was transferred to a plate containing NGM seeded with OP 50 bacteria. After absorption of the liquid into the agar animals were picked with a platinum wire into the microcompartments.

### Behavioral observations and calcium measurements

Filming in microcompartments is described in reference [19]. For screening and bout analysis we used time-lapse movies with 5s interval. A bout of immobility was defined as no detectable movement lasting at least 5s. To precisely measure nose speed we used burst type protocols: every 15min we took 40 frames with an interval of 0.5s. Nose speeds were found empirically to be a sensitive measure for quiescence. Both dorsoventral head movements known as foraging and locomotion contributed to nose speed. The spatial resolution of the images was 0.4 µm per pixel. A person that was not blind to the phenotype did the manual tracking of the worm nose using Andor IQ2 software. For averaging data from burst movies, we pooled all burst movies collected during lethargus for each individual worm before averaging across different individuals. The averaged data from one pool of burst movies obtained from one individual animal was regarded as one N. Similarly, we pooled burst movies collected during wake-like behavior for each individual worm before averaging across different individuals. The period of wake-like behavior was much longer than the lethargus period. Thus, we did not use the entire L1 wake-like period for analysis but selected the last ten bursts that were preceding the sleep-like phase. We raised all worms at a temperature of 15°C. The microscope room was temperature-controlled to 19°C using air conditioning. During movie acquisition, we used a lid to prevent drying of the hydrogel microcompartment. The lid was heated to 25.5°C to prevent fogging. The culture temperature was measured with a pt1000 resistance thermometer and was 23°C. After placing pretzel stage eggs of N2 or *egl-30*(*tg26gf*) mutants into the microcompartments, we waited four to six hours before we started data acquisition. Because *egl-30*(*n715sd*) mutants were egg laying defective and larvae developed more slowly than wild type, we cut open gravid hermaphrodite worms in a drop of S-Basal and placed the eggs onto a fresh NGM plate 12h before preparing the microcompartments. We then transferred freshly hatched L1 larvae into the microcompartments. In addition to staging worms according to their age after hatching, the cuticles of larvae were checked for L1-specific alae. To identify lethargus, we manually scored pharyngeal pumping in bright field or DIC movies acquired at each data point. Shedding of the cuticle was visually identified, and followed each lethargus period. Complete shedding of the cuticle was defined as the completion of the molt. While the growth rate of *egl-30*(*n715sd*) worms was reduced, we were able to clearly identify L1 lethargus in every individual worm based on the above criteria. No worms were censored due to error in picking them at the right stage of lethargus. N2 and *egl-30*(*tg26gf*) worms were kept inside microcompartments and were imaged for 20h. *egl-30*(*n715sd*) mutants were filmed for 40h. 10% of *egl-30*(*n715sd*) worms arrested or died during the experiment and did not progress through the molt. We discarded the data for these animals. For aligning the data from several animals, the beginning of pumping cessation was used and was arbitrarily set to 0h. Completion of cuticle shedding was defined as the “molt” in plots. The lethargus was defined as the period of continuous absence of pumping lasting at least 1.5h, followed by shedding of the cuticle. Pumping was scored by visually scoring pharyngeal pumping and pharyngeal structure that appears clear during lethargus. Neural GCaMP3.35 expressing animals, calcium measurements and mechanical stimulation are described in reference [4]. For determining the maximum amplitude of the stimulus-induced calcium transient, first the amplitude for each individual worm was determined and then data from several worms were averaged. Because the maximum of the transient occurred at slightly different times after stimulation in different individual worms, the calculated maximum amplitude appeared slightly higher than the maximum amplitude on the plot showing the averaged data for each time point. For quantification of GCaMP3.35 signals we computed ΔF/F values. F was calculated as the normalized average fluorescence signal of the 2.5h preceding lethargus. Waking intensity before stimulation was used as F for evoked neural activity. Pan-neural signals were analyzed by cutting out a region of interest containing the head region. ALM signals were analyzed by cutting out a small region of interest containing the neuron. As background served a region that did not contain neural signal. For analysis of evoked calcium transients we looked at intensity signals in PLM, because it was easier to keep PLM in focus. Muscle GCaMP3.35 calcium measurements and curvature analysis are described in reference [5].

### Rescue construct

We made a synthetic *mkate2*-tagged version of *egl-30*. We amplified the putative promoter and 3’ untranslated region of *egl-30* using the following primers:

Promoter
Fwd: GGGG ACA ACT TTG TAT AGA AAA GTT GCC GAA AAC AAT GGA AAG AAG CAT TA
Rev.: GGGG AC TGC TTT TTT GTA CAA ACT TGG GCG GCC GAA AAG GTG CCA C
UTR
Fwd: GGGG ACA GCT TTC TTG TAC AAA GTG GGA AGA AGT CGC ATG TCG GAT TG
Rev.: GGGG AC AAC TTT GTA TAA TAA AGT TGG CAT TTAG CTA TAT CAG AAA GCA C

For cloning we used the Multi-Site Gateway Three-Fragment Vector Construction Kit (Invitrogen). After bombardment into *unc-119*(*ed 3*), *goeIs42* (as well as the other rescue insertions) was first backcrossed two times in to N2 and was then crossed into *egl-30*(*n715sd*) afterwards using the hT2 balancer. For muscle and pan-neuronal rescue we obtained more than one transgenic line. We averaged the data from the different transgenic lines: For pan-neuronal rescue we pooled data from HBR599(N = 10) and HBR600(N = 6) and for muscle rescue we pooled data from HBR569(N = 8), HBR602(N = 6), and HBR604(N = 6).

### Channelrhodopsin experiments

All trans retinal (ATR, Sigma) was added to the sample. ATR was soaked into the agarose after its solidification. For control experiments that did not contain retinal, only vehicle (50% ethanol) was soaked into the agar. The volume of the agar was 1ml. We added 10µl of ATR solution to the sample so that the final concentration was 250µM. The final concentration of ethanol was 0.5%. Blue light (100 pulses with 15ms duration with an interval of 70ms) was delivered every 30 minutes using a 495nm LED (CoolLED) coupled to the fluorescence port of the microscope. LED power was measured with a light voltmeter. LED intensity scaled approximately linearly with light density. 10% LED intensity corresponded to 0.5mW/mm^2^. We performed the experiment with LED intensities of 0%, 2%, 5%, 10% 20%, 50%, and 100%. Backward movement of animals was scored manually. A worm was scored as responding, if it moved at least 5µm backwards upon stimulation. Because the movement of *egl-30*(*n715sd*) animals was uncoordinated, we scored the presence or absence of backwards movement but not the quality of backwards movements. Each worm was tested four (wild type and *egl-30*(*tg26gf*)) to eight (*egl-30(n715sd*)) times during each sleep-like and wake-like behavior. The measurements for both sleep-like and wake-like behavior were averaged for each individual worm to obtain one N. The experiment was performed with N = 5 worms for each LED condition and genotype. Half-maximum responsiveness was interpolated using Origin software.

### Statistical tests and data display

For statistical analysis all data points during the sleep-like states were averaged and compared with averaged data points of the beforehand wake-like state (2-2.5 hours). For statistical tests comparing sleep-like and wake-like behavior, Wilcoxon Signed Rank tests (two tailed) were performed using Origin software. For statistical tests comparing mutants with wild type t-tests were performed using Origin software. Analysis of Variance for Simple Linear Regression (ANOVA, Figure 1A) was used for testing the correlation of wake-like and sleep-like activity in different strains. We used Iglewicz and Hoaglin’s robust test to test the reduction of velocity during sleep-like state compared to wake-like state for multiple outliers. Values were considered as potential outliers, when the modified Z-score was greater than 3.5 [20]. For displaying micrographs we applied false colors to the grayscale image with the standard color map “jet” in Matlab.

## Results

### Wake-like and sleep-like activity in mutants with altered activity levels

By definition, a sleep-like state is separated from a wake-like state based on differences in behavioral activity. Behavioral activity is massively reduced during the sleep-like state relative to the wake-like state [1,2,6,12,19]. Intuitively, increased activity during wake-like behavior is expected to also cause increased activity during sleep-like behavior: publications describing mutants with altered sleep or sleep-like behavior often also contain measures of general activity levels to assay the specificity of the mutation. Whereas hyperactivity during wake often correlates with decreased sleep, some mutants have been found that have reduced sleep but normal activity levels during wake [2,11,12,21,22]. Using a similar argument, one may expect mutants with generally decreased activity levels during wake-like behavior to also have decreased activity during sleep-like behavior.

In *C. elegans*, mutants have been found that have generally altered behavioral activity levels [23]. We wanted to test whether hyperactivity is typically associated with reduced sleep-like behavior and whether hypoactivity is typically associated with increased sleep-like behavior. Rather than looking at absolute behavioral activity measures of mutants, we compared their sleep-like behavior with their wake-like behavior and selected those mutants that displayed little difference between these behaviors. We selected an arbitrary and non-exhaustive set of strains that were already described to have altered behavioral activity levels (Figure 1) [10,13,17,23-37]. To analyze these mutants, we filmed the first wake and sleep-like cycle inside microfluidic culture compartments with a frame rate of 0.2 per second and quantified movement by measuring nose speed [19]. We identified L1 lethargus based on two criteria: firstly, pumping cessation lasted at least 1.5h and secondly, shedding of the cuticle followed pumping cessation. Because all of the strains showed cessation of pumping and went through the molt, we could identify lethargus in all mutant strains using these criteria. We compared nose speed during the wake-like state with nose-speed during the sleep-like state. We found that nose speed was strongly reduced during sleep-like behavior in most mutants. The average relative nose speed during the sleep-like state was 60% of the wake-like state. Nose speed during wake-like behavior generally correlated with nose speed during sleep-like behavior (Pearson’s R: 0.79, p < 0.001, ANOVA).

In three mutants, activity levels during sleep-like behavior were higher than 80% of the wake-like state, and were significantly different from wild type according to a two-sample t-test (p < 0.01, still significant after Bonferroni correction). Among these mutants were two hyperactive mutants and one severely hypoactive mutant. One of the hyperactive genes was a hypermorphic gain-of-function allele of *egl-30*(*tg26gf*) [17]. The other one was a complete-loss-of-function allele of *goa-1*(*sa734*) [24]. The hypoactive mutant was an allele of the conserved heterotrimeric G protein alpha q subunit gene *egl-30*(*n715sd*) [13,16]. *egl-30*(*n715sd*) was originally described as a semidominant allele that may be antimorphic: Like *egl-30* loss-of-function alleles, *egl-30*(*715sd*) showed reduced locomotion and egg-laying. Worms that were heterozygous for *egl-30*(*715sd*) also showed reduced locomotion and egg-laying, but the phenotypes were less severe than in homozygous mutants. Thus, *egl-30*(*n715sd*) may not be a typical hypomorphic loss-of-function allele [15,16]. *egl-30*(*n715sd*) and *goa-1*(*sa734*) were significant outliers according to Iglewicz and Hoaglin’s robust test [20]. However, the interpretation of these values as outliers is difficult because they are at the extreme ends of the population.

The findings from this analysis suggested that nose speed during wake and sleep-like behavior typically correlated: increased movement during wake-like behavior was associated with increased movement during sleep-like behavior in most mutants. However, we found one hypoactive mutant with less pronounced activity differences between wake and sleep-like behavior. These results suggest a role for *egl-30* in regulating activity differences between wake-like behavior and sleep-like behavior. *egl-30* is a known regulator of neural function and also of many behaviors such as pharyngeal pumping and locomotion [13].

We further characterized wake-like and sleep-like behavior in both the semidominant hypoactive *egl-30*(*n715sd*) mutant as well as the hypermorphic hyperactive *egl-30*(*tg26gf*) mutant. We could not study an *egl-30* null allele, because complete-loss-of-function of *egl-30* is lethal [13].

### *egl-30* mutants have reduced sleep-like immobility

**Figure 2:**
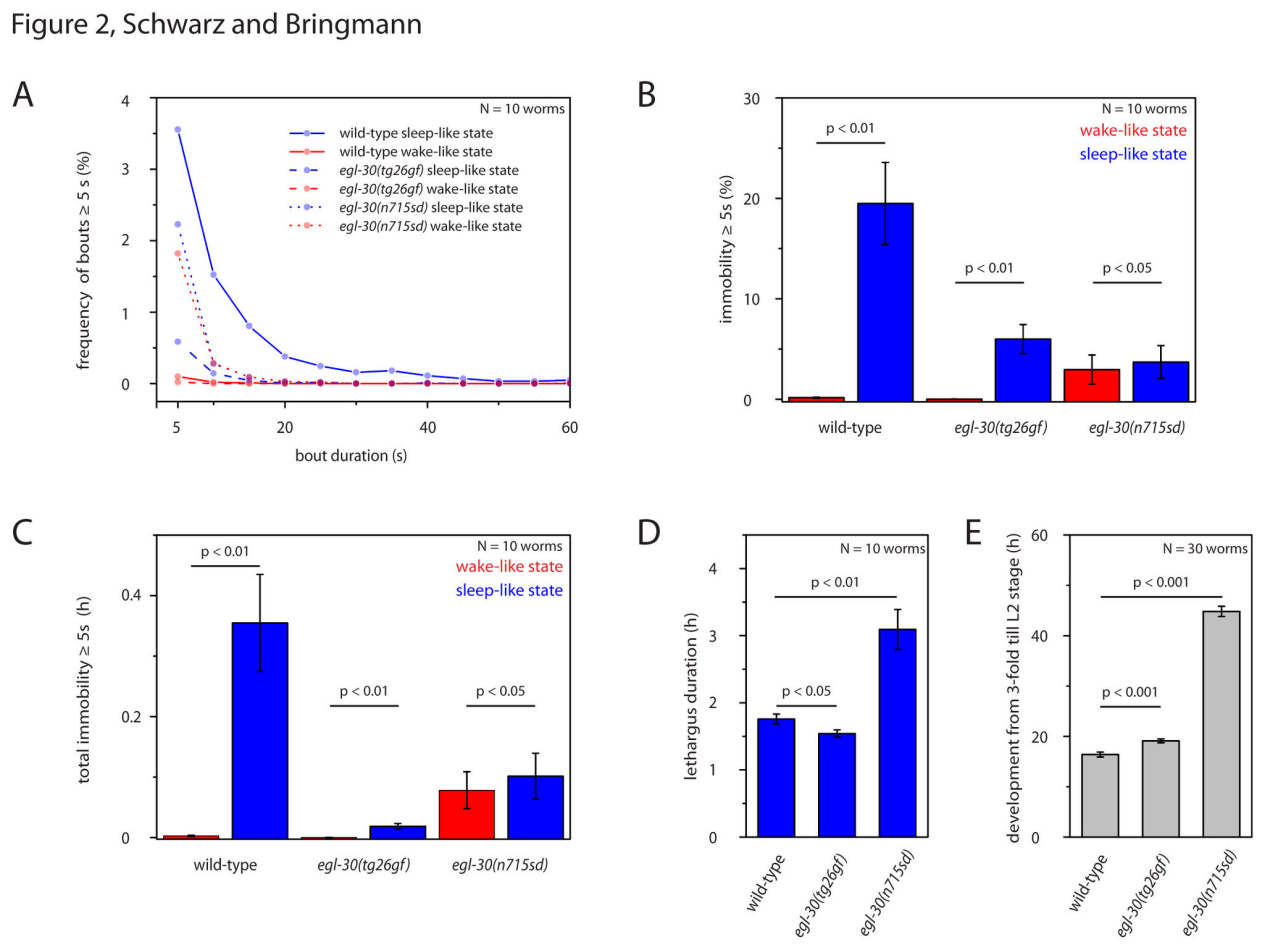
Quiescence bout lengths in *egl-30* mutants. **A** Frequency distribution of quiescence bout durations for wild type and *egl-30* mutants calculated as the fraction of lethargus length (or a time window of 2h during wake-like behavior). A bout was defined as immobility lasting 5s or longer. **B** Quantification of total immobility ≥ 5s calculated as a fraction of lethargus length (or a time window of 2h during wake-like behavior). **C** Cumulative immobility ≥ 5s integrated over the entire lethargus phase (or a time window of the same length during wake-like behavior). D Mean lethargus duration in hours as defined by the absence of pharyngeal pumping. E Mean development time in hours from 3-fold embryo until L2 larvae in wild type and *egl-30* mutants. Error bar represent SEM. A paired sample Wilcoxon test was used for statistical testing of wake-like versus sleep-like behavior. T-test was used for comparing different genotypes.

Complete immobility is a hallmark of sleep-like quiescence during lethargus in *C. elegans* [2,3]. The nose moved constantly during wake-like behavior, even when the worms were dwelling and did not show long-range locomotion. We thus quantified nose immobility as an approximation for complete immobility: Immobility was defined as no detectable movement of the nose. During wild type sleep-like behavior, larvae were totally immobile for 19% of the sleep-like behavior and bouts of complete quiescence lasted on average 15s (Figure 2A,B). During sleep-like behavior in hyperactive *egl-30*(*tg26gf*) mutants, full immobility was seen for only 6% of the sleep-like behavior and bouts of complete quiescence lasted on average only 7s (Figure 2A,B). In hypoactive *egl-30*(*n715sd*) mutants, immobility was present only 4% of the sleep-like behavior and bouts of complete quiescence lasted only 6s (Figure 2A,B). Because lethargus and the general development of *egl-30*(*n715sd*) lasted twice as long as wild type or *egl-30*(*tg26gf*) (Figure 2D,E), we integrated immobility over the entire lethargus period. The integrated immobility of *egl-30* mutants was also substantially reduced compared with wild type (Figure 2C). Thus, complete immobility was severely reduced in *egl-30* mutants.

### Nose speed distribution in *egl-30* mutants

*egl-30* loss-of function mutants generally move very little and we wanted to quantify the small movements of the nose as precisely as possible. We thus filmed the *egl-30* mutants again and increased the frame rate to two per second. To reduce data amounts to a manageable amount, we filmed the worms discontinuously using a burst mode every 15 minutes for twenty seconds. Probably, burst-type measurements were more precise than the five-second-interval protocol because it less likely missed small movements. We calculated a probability distribution for all nose speeds during sleep-like and wake-like behavior.

**Figure 3:**
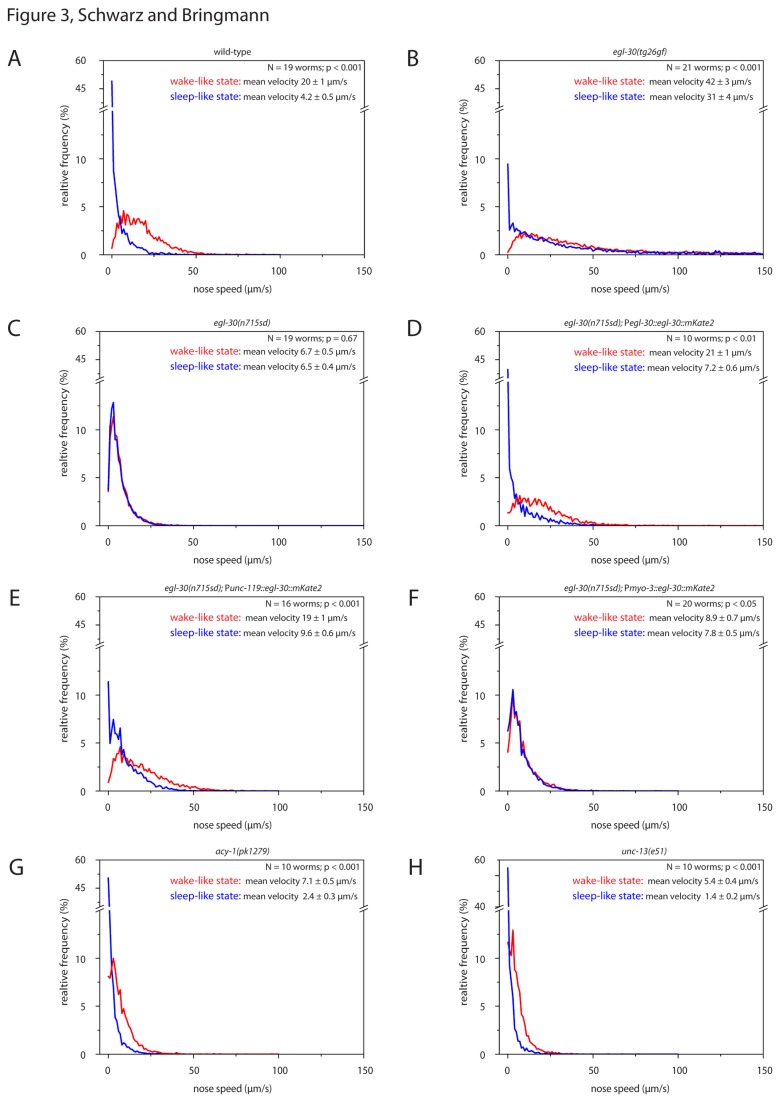
Probability distribution of nose speed in *egl-30* mutants, *egl-30* rescue mutants and hypoactive mutants during wake-like and sleep-like behavior. Probability distribution of nose speeds during wake-like and sleep-like state. **A** wild type, **B** *egl-30*(tg26gf), **C** *egl-30*(n715sd), **D** *egl-30*(n715sd) transformed with a rescue construct expressed from its endogenous promoter, **E** *egl-30*(n715sd) transformed with a pan-neural rescue construct, **F** *egl-30*(n715sd) transformed with a muscle rescue construct, **G** *acy-1*(pk1279lf) and **H** *unc-13*(e51lf). We can exclude that the rescue of *egl-30*(n715sd) was caused by the *myo-3* or *unc-119* promoter regions, because transgenic worms that contained these promoters without the *egl-30* *cDNA* such as *pmyo-3::gcamp* (Figure 4) or *punc-119::gcamp* (Figure 5) did not show rescue (Figure 4, 5 and data not shown). Complete immobility of the nose was virtually absent in *egl-30* mutants, rescued by the *egl-30* transgene and partially rescued by the pan-neural rescue construct. Values displayed show mean nose speed ± SEM. A paired sample Wilcoxon test was used for statistical testing of wake-like versus sleep-like behavior.

During wild type sleep-like behavior, average nose speed was reduced by 78% compared with wake-like behavior (Figure 3A). During sleep-like behavior in hyperactive *egl-30*(*tg26gf*) mutants, worms reduced their movement by only 26% (Figure 3B). Average nose speed during sleep-like behavior in hypoactive *egl-30*(*n715sd*) mutants was reduced by only 3% (Figure 3C). Nose speed in *acy-1*(*pk1279*) and *unc-13*(*e51*) was clearly reduced during sleep-like behavior, indicating that nose speed reduction can be detected in hypoactive mutants (Figure 3G,H). The statistics for this experiment can be found in Figure S1. The more precise nose speed measurement showed that during sleep-like behavior, hypoactive *egl-30*(*n715sd*) mutants have higher nose mobility than wild type.

### Rescue of the *egl-30* semidominant phenotype

We wanted to test whether the sleep-like state phenotype in *egl-30*(*n715sd*) was actually caused by the mutation in *egl-30* or by a background mutation. To do this we tested for rescue of the mutant phenotype using an *mkate2*-tagged version of *egl-30*. *mkate2* encodes a far-red fluorescent protein [38] and was codon-optimized for expression in *C. elegans* using *codon adapter* [39]. The tagging was done according to an established strategy into an internal loop of the G protein [40]. The insertion site was analogous to that of a published, functional *gfp*-tagged *goa-1* transgene [41]. *egl-30*(*n715sd*) mutants carrying the *egl-30::mkate2* rescue construct showed a clear reduction of nose speed during sleep-like behavior of 66% compared with the wake-like behavior and pronounced immobility during sleep-like behavior (Figure 3D). Because the *egl-30::mkate2* transgene rescued the *egl-30*(*n715sd*) phenotype we conclude that the phenotype is actually caused by *egl-30*(*n715sd*).

*egl-30* is expressed in both muscle and neurons [13]. To test in which tissue expression of *egl-30* is required for sleep-like state control, we expressed the *egl-30::mkate2* transgene in either body wall muscles using a *myo-3* promoter or in the entire nervous system using an *unc-119* promoter [42,43]. *egl-30*(*n715sd*) mutants carrying the *punc-119::egl-30::mkate2* rescue construct showed a strong rescue in activity levels, and also a clear reduction of nose speed during sleep-like behavior of 49% (Figure 3E). *egl-30*(*n715sd*) mutants carrying the *pmyo-3::egl-30::mkate2* rescue construct showed an increase in general activity levels, but only showed a small reduction of nose speed during sleep-like behavior of 12% (Figure 3F). The statistics for this experiment can be found in Figure S1. Thus, we could partially rescue the locomotion and sleep-like phenotype of *egl-30*(*n715sd*) by expressing *egl-30* only in the nervous system, and we could partially rescue the locomotion phenotype but not the sleep-like state phenotype by expressing *egl-30* only in muscle. We conclude that *egl-30* acts mostly in the nervous system to control sleep-like behavior.

### Reduced muscle relaxation in *egl-30* mutants during sleep-like behavior

**Figure 4:**
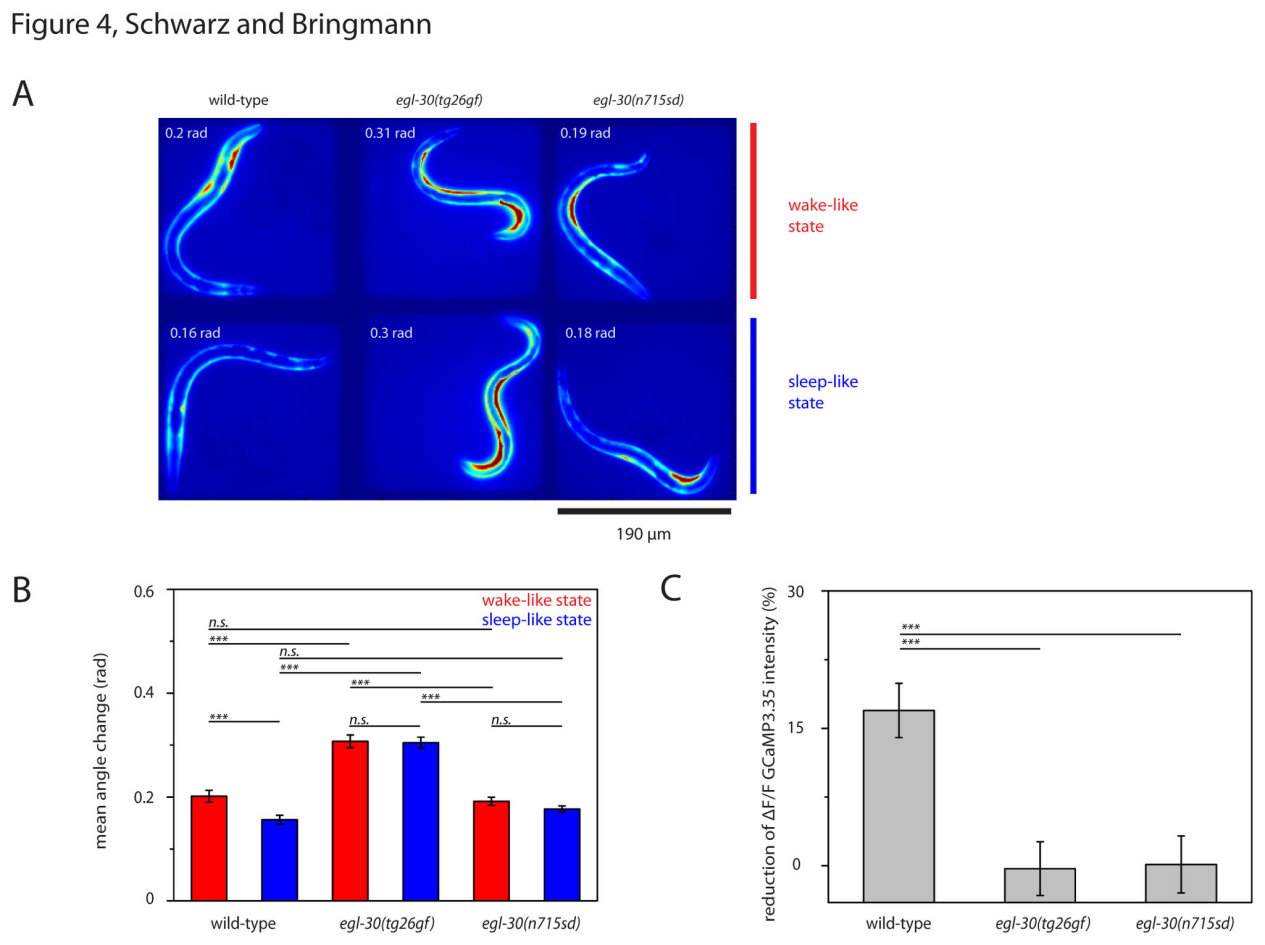
Reduced muscle relaxation in *egl-30* mutants during sleep-like behavior. **A** False color fluorescence images of wild type and *egl-30* mutants expressing GCaMP3.35 in body wall muscle during the wake-like state (top) and during the sleep-like state (bottom). Bright signals reflect muscle activity. The angle change is displayed for each example worm. B Mean angle change in wild type and *egl-30* mutants during wake-like and sleep-like state. **C** Reduction of muscle calcium signals in wild type and *egl-30* mutants. Note the reduction of angle change and muscle calcium during sleep-like behavior in wild type. Body curvature and muscle calcium are much less reduced in *egl-30* mutants. Error bars represent SEM. For each strain ≥ 10 animals were tested (two-sample t-test for comparisons between different genotypes, paired sample Wilcoxon test for comparison of wake-like and sleep-like behavior, *** denotes statistical significance at p<0.001)..

During lethargus sleep-like behavior, *C. elegans* reduce their body wall muscle activity and assume a relaxed posture [5,6]. Do *egl-30* mutants also relax their muscles and assume a typical posture? To measure muscle activity we expressed GCaMP3.35 in striated body wall muscle cells and filmed worms during wake and sleep-like behavior (Figure 4A). We quantified GCaMP3.35 intensity and quantified postures by measuring the angle change along the body axis. We found that during wild type lethargus, GCaMP3.35 **Δ**F/F was reduced by 17% and that average angle change was reduced by 20% (Figure 4B,C). During *egl-30*(*tg26gf*) lethargus, GCaMP3.35 **Δ**F/F was not reduced and average angle change was reduced by only 3% (Figure 4B,C). During *egl-30*(*n715sd*) lethargus, GCaMP3.35 **Δ**F/F was reduced by only 1% and average angle change was reduced by 5% (Figure 4B,C). This indicated that *egl-30* mutants relaxed their muscles less during lethargus and assumed a less pronounced sleep-specific elongated posture compared with wild type.

### Reduced decrease of spontaneous neural activity in *egl-30* mutants during sleep-like behavior

**Figure 5:**
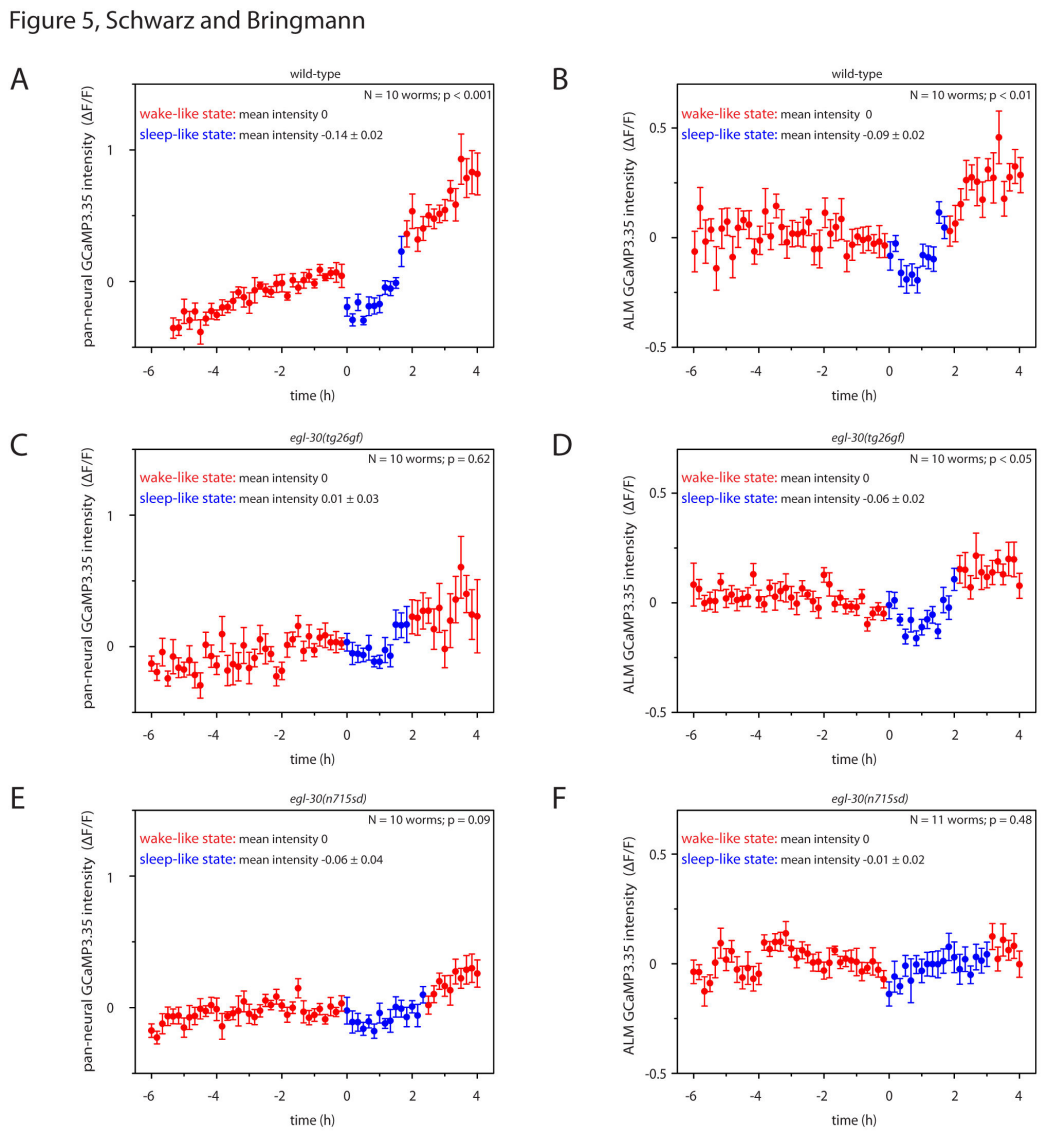
Reduced decrease of spontaneous neural activity in *egl-30* mutants during sleep-like behavior. **A** Spontaneous pan-neural activity was reduced during wild type lethargus. **B** Spontaneous ALM activity was reduced during wild type lethargus. **C** Spontaneous pan-neural activity and D ALM activity in *egl-30*(tg26gf) was reduced less than in wild type during lethargus. **E** Spontaneous pan-neural activity and F ALM activity in *egl-30*(n715sd) was reduced less than in wild type during lethargus. Error bars represent SEM. The beginning of lethargus was arbitrarily set to 0h. Baseline fluorescence during the two hours of wake-like behavior preceding lethargus was set to zero. Statistical test used was Wilcoxon Signed Ranks.

During *C. elegans* lethargus, spontaneous neural activity is reduced globally as well as in individual sensory neurons [4,6]. How does spontaneous neural activity change during lethargus in *egl-30* mutants? We measured neural activity using transgenic animals expressing GCaMP3.35 either in all neurons or in mechanosensitive neurons and quantified GCaMP3.35 intensity. During lethargus in wild type animals, pan-neural GCaMP3.35 **Δ**F/F was reduced by 14% and mechanosensitive neuron GCaMP3.35 **Δ**F/F was reduced by 9% (Figure 5 A, B). During lethargus in *egl-30*(*tg26gf*) mutants, global GCaMP3.35 intensity was not measurably reduced and ALM intensity was reduced by only 6% (Figure 5 C, D). During lethargus in *egl-30*(*n715sd*) mutants, global GCaMP3.35 intensity was reduced by only 7% and ALM intensity was reduced by only 1% (Figure 5 E, F). We conclude that spontaneous neural activity in *egl-30* mutants was less reduced during lethargus compared with wild type.

### Responsiveness of the nociceptive ASH circuit in *egl-30* mutants during sleep-like behavior

During sleep-like behavior in *C. elegans*, responsiveness to stimulation is reduced [2]. Nociceptive stimulation is sensed mainly by the polymodal ASH neuron [44]. Optogenetic activation of ASH causes backwards movement [45]. How is the responsiveness of the ASH nociceptive circuit altered in *egl-30* mutants? We optogenetically stimulated ASH and measured the behavioral response. We used transgenic worms that expressed Channelrhodopsin2 under the control of the *sra-6* promoter. This promoter drives strong expression in ASH but also in PVQ and weakly in ASI [46]. The backwards movement that is induced by blue-light stimulation of *psra-6::Channelrhodopsin2*-expressing animals was shown to be caused by activation of ASH [45,47].

Worms were cultured inside microfluidic microcompartments from the early L1 stage until the L2 stage and were filmed repeatedly using a burst protocol similar to the burst protocols described above. Short movies were collected every 30 minutes. During each burst movie, worms were exposed to blue LED light of a defined intensity. Movies were analyzed by manually scoring backwards movement. In order to obtain a dose response relationship for backwards movement and LED intensity, the experiment was repeated several times with different LED intensities. For each LED intensity a set of different individual worms was assayed. The fraction of larvae responding generally increased with LED intensity. We plotted the fraction of events in which worms showed backwards movements during LED illumination against the LED intensity and used the resulting dose response curve to estimate the LED intensity required for backwards movement in 50% of illumination events. We defined this value as the response threshold of the nociceptive ASH circuit.

**Figure 6:**
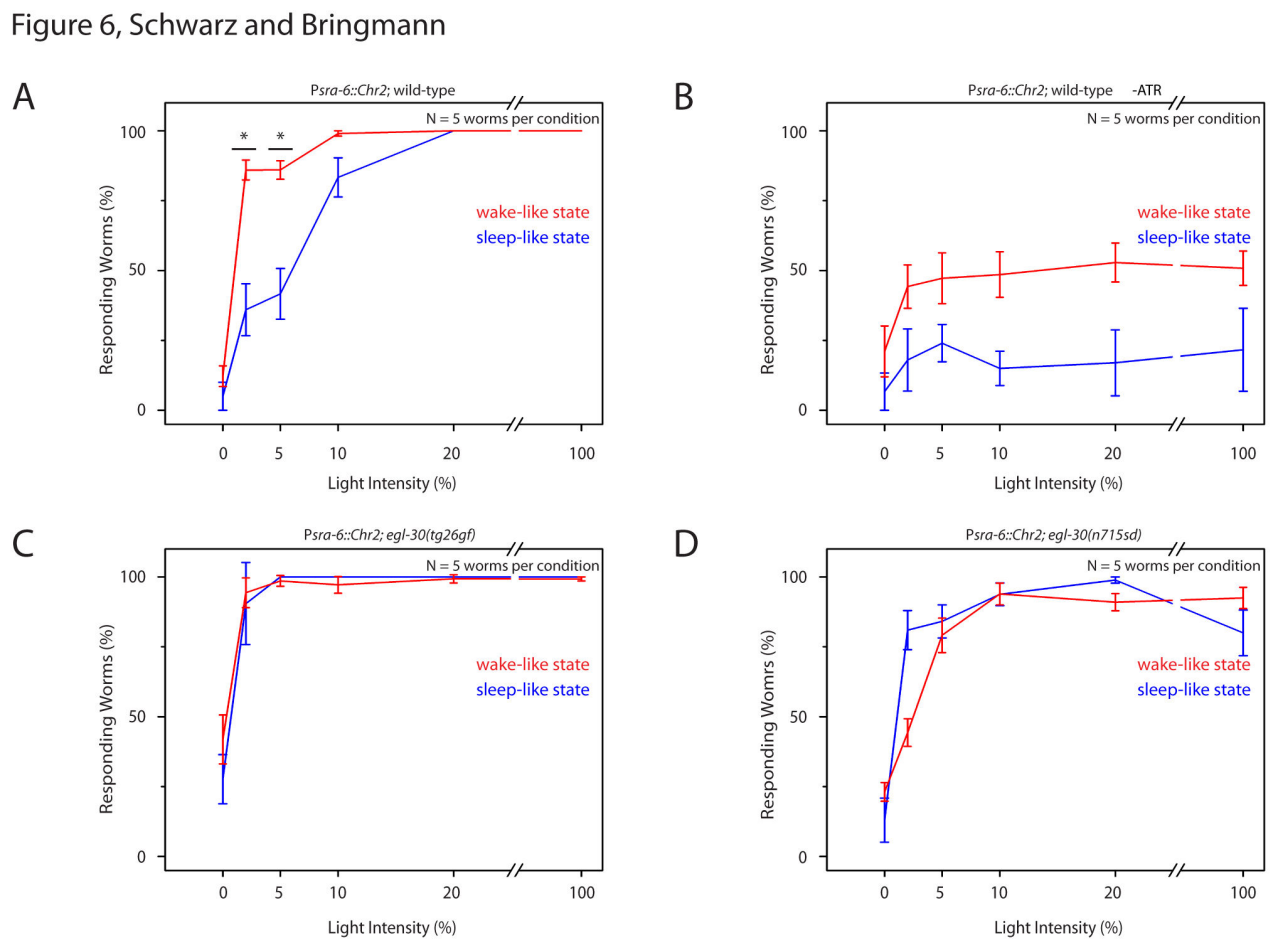
Reduced decrease of sensory responsiveness during sleep-like behavior in *egl-30* mutants. **A** Light dose-dependent fraction of wild type worms reversing upon optogenetic ASH activation. Note that worms need higher LED intensity for half-maximal responsiveness during sleep-like behavior. B Wild type control without retinal. **C** Light dose-dependent fraction of worms reversing upon ASH activation during wake-like and sleep-like states in *egl-30*(tg26gf). **D** Light dose-dependent fraction of worms reversing upon ASH activation during wake-like and sleep-like states in *egl-30*(n715sd). Note that the intensity required for half-maximal responsiveness is not reduced during lethargus in *egl-30* mutants. Error bars represent SEM. * represents p < 0.05, Wilcoxon Signed Ranks Test.

During wake-like behavior in wild type the response threshold was 1%. During sleep-like behavior in wild type the response threshold was 6%. At LED powers of 20% or higher, larvae during both wake and sleep-like behavior responded. Thus, during sleep-like behavior, the response threshold was increased (Figure 6A). Control animals that were raised without all-trans-retinal also showed some responsiveness to the LED stimulation, albeit much less than in the presence of all-trans-retinal (Figure 6B). *egl-30*(*tg26gf*) mutants were generally more sensitive to stimulation: During wake-like behavior the response threshold was 0.3%, during sleep-like behavior the response threshold was 0.7% (Figure 6C). *egl-30*(*n715sd*) mutants were less sensitive to stimulation: During wake-like behavior the response threshold was 2%, during sleep-like behavior the response threshold was 1% (Figure 6D).

We tested response thresholds to aversive stimulation using blue light-induced Channelrhodopsin2 activation. The majority of this stimulation is likely to be caused by ASH activation but also included blue-light avoidance [48]. While responsiveness to aversive stimulation was reduced during sleep-like behavior in wild type, responsiveness was not reduced during sleep-like behavior in *egl-30* mutants.

### Reduced decrease of responsiveness to mechanical stimulation in *egl-30* mutants during lethargus

**Figure 7:**
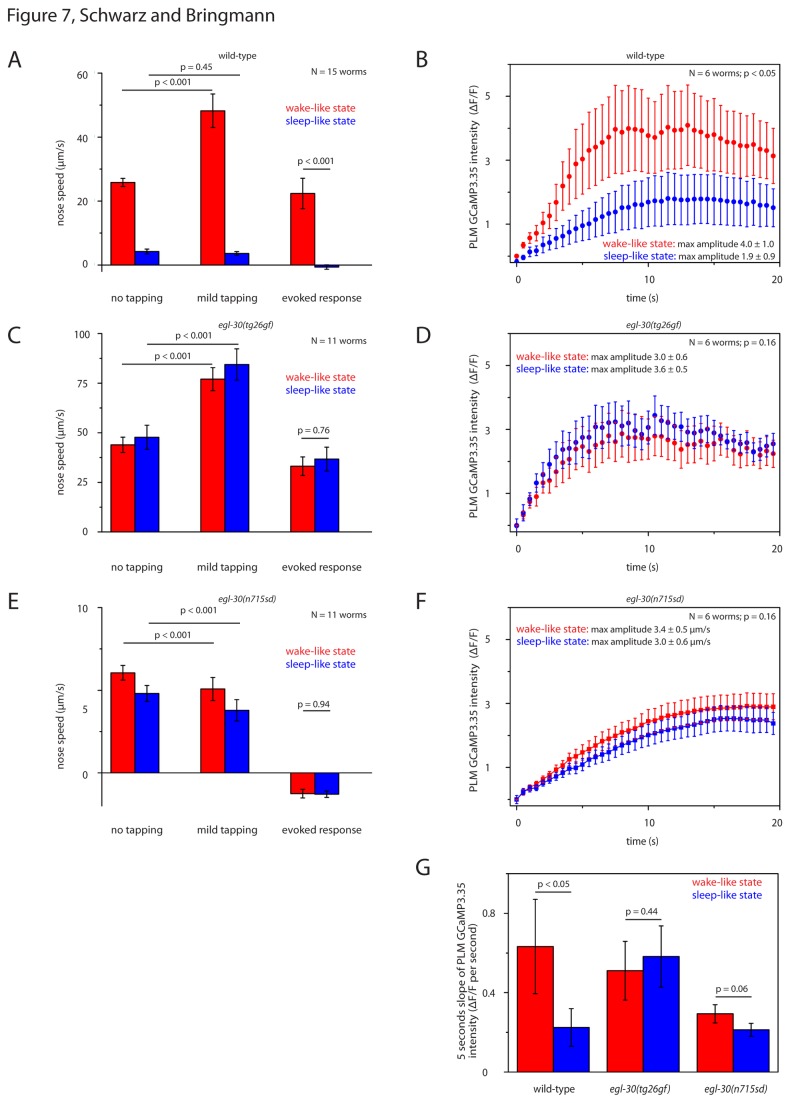
Reduced decrease of responsiveness to mechanical stimulation in *egl-30* mutants during lethargus. **A** Evoked dish tapping-induced behavioral response was reduced during wild type lethargus. “evoked response” was defined as nose speed during “mild tapping” minus nose speed before tapping (“no tapping”). **B** Dish tapping-induced calcium transients in mechanosensory neurons were reduced during wild type lethargus. **C** Evoked behavioral response to dish tapping in *egl-30*(tg26gf) mutants was not reduced during sleep-like behavior. Compared with wild type, the evoked response of *egl-30*(*tg26gf*) was not reduced during wake-like behavior, but was significantly reduced during sleep-like behavior (p < 0.001, t-test). **D** Evoked calcium transients in mechanosensory neurons were not reduced during lethargus in *egl-30*(tg26gf). **E** Evoked behavioral response to dish tapping in *egl-30*(n715sd) mutants was not reduced during sleep-like behavior. Compared with wild type, the evoked response of *egl-30*(*n715sd*) was not reduced during sleep-like behavior, but was significantly reduced during wake-like behavior (p < 0.001, t-test). **F** Evoked calcium transients in mechanosensory neurons were less reduced during lethargus in *egl-30*(n715sd). **G** Slope of the tapping-induced calcium transient in mechanosensory neurons during the first five seconds. While the initial slope was significantly decreased during sleep-like behavior in wild type (paired sample Wilcoxon test), the difference between wild type and *egl-30* mutants was not statistically significant.

Upon gentle mechanical stimulation, *C. elegans* typically first undergo a short rapid backwards movement [49]. During the backwards movement, dorsoventral nose speed is reduced [50]. During lethargus, *C. elegans* have reduced responsiveness to gentle mechanical stimulation [2]. Is reduced responsiveness to stimulation during lethargus altered in *egl-30* mutants? We stimulated worms using dish tapping and filmed their behavior before and after stimulation. We cultured mutant and wild type worms in microcompartments. Every 30 minutes we filmed the animals for 20 seconds, then started dish tapping, and filmed the response for another 20 seconds. During the wake-like state, after stimulation, wild type larvae showed the typical early backwards response and suppression of head movements. Stimulation increased nose speed by 87%. During the sleep-like state, after stimulation, wild type larvae almost never responded with a backwards movement. If the nose was slowly moving during lethargus, stimulation typically caused a reduction of this movement. Thus, during lethargus stimulation average nose speed even decreased by 14% (Figure 7A). During wake-like behavior, upon stimulation, *egl-30*(*tg26gf*) larvae showed the typical early backwards response and suppression of head movements. Stimulation increased nose speed by 75%. During lethargus, upon stimulation, *egl-30*(*tg26gf*) showed a backwards movement that was indistinguishable from the wake-like state: Stimulation increased nose speed by 76% (Figure 7C). *egl-30*(*n715sd*) larvae did not show a stimulus-induced backward response. However, upon stimulation, they showed a suppression of lateral head movements. During wake-like behavior, stimulation decreased nose speed by 16%. During lethargus, stimulation decreased nose speed by 21% (Figure 7E). We conclude that *egl-30*(*tg26gf*) mutants always responded with a typical backwards movement to stimulation during both wake and lethargus. *egl-30*(*n715sd*) mutants always responded to stimulation, albeit with a limited behavioral response. The different behavioral response makes a direct comparison with wild type difficult. Nevertheless, these results are consistent with the idea that behavioral differences between wake and lethargus are reduced in these mutants.

### Reduced decrease of excitability of a mechanosensory neuron in *egl-30* mutants during the sleep-like state

Mechanosensory neurons show a decrease in excitability during lethargus, which corresponds to a decrease in behavioral responsiveness [4]. Is excitability of mechanosensory neurons altered during lethargus in *egl-30* mutants? We mechanically stimulated animals during wake and lethargus and measured evoked calcium transients in PLM. We cultured individual worms expressing GCaMP3.35 in microcompartments. Every 30 minutes we stimulated worms by dish tapping and filmed GCaMP3.35 intensity. During lethargus in wild type worms, stimulus-evoked calcium transients were reduced by 53% (Figure 7B,G). During lethargus in *egl-30*(*tg26gf*) mutants, transients were not reduced (Figure 7D,G). During lethargus in *egl-30*(*n715sd*) mutants, transients were decreased by only 13% (Figure 7F,G). Taken together, stimulation-induced calcium transients were much less reduced during lethargus in *egl-30* mutants compared with wild type.

## Discussion

Surprisingly, we found a severely hypoactive *egl-30*(*n715sd*) mutant that is less immobile during the developmental time sleep-like behavior should occur. Compared with wild type wake-like behavior, *egl-30*(*n715sd*) larvae moved very little, and their behavioral and neural responsiveness to stimulation was generally low. The locomotion behavior and sensory responsiveness of *egl-30*(*n715sd*) did not change dramatically between lethargus and wake-like behavior.

Both the gain-of-function and the semidominant *egl-30* mutants had defects in sleep-like behavior. We do not know why *egl-30* mutations result in these complex defects. We can only speculate that *egl-30* mediates activating, wake-promoting signaling pathways as well as inhibiting, sleep-promoting signaling pathways.

Additional alleles of *egl-30* exist. There are several alleles, including potential null alleles, that produce stronger loss-of-function than *egl-30*(*n715sd*) and that result in embryonic lethality. There are several hypomorphic loss-of-function alleles that produce weaker loss-of-function than *egl-30*(*n715sd*) judged by their egg-laying and movement phenotypes [13]. We have not characterized any of these mutants in detail because these loss-of-function mutants clearly displayed lethargus quiescence when visually inspected on an NGM plate. Loss-of-function alleles of *egl-30* that we inspected and that showed quiescence were *egl-30*(*n686*), *egl-30*(ad805), and *egl-30*(ad806).

*egl-30*(*n715sd*) lack a splice consensus site which results in an EGL-30 protein that is lacking its C-terminus [13]. An intragenic suppressor mutation of *egl-30*(*n715sd*), *egl-30*(*n715n1190*), rescues the egg-laying phenotype and has a new splice site which results in a protein that has a small deletion but a normal C-terminus [13]. We found that *egl-30*(*n715n1190*) also partially rescued the sleep-like behavior phenotype (nose speed during wake-like behavior was 9.4 µm/s and nose speed during sleep-like behavior was 5.2 µm/s, N = 10). Thus, *egl-30*(*n715sd*) appears to be a fairly unusual allele: it is the strongest available hypoactive allele of *egl-30* that still produces viable offspring. We do not know why *egl-30*(*n715sd*) has this unusual phenotype. One hypothesis would be that the C-terminal part of *egl-30*, which is lacking in *egl-30*(*n715sd*), is specifically required for sleep-like behavior control. The truncated protein could inappropriately interact with other molecules. These inappropriate interactions could be masked if normal *egl-30* is in excess during rescue experiments. A different hypothesis would be that *egl-30* loss-of-function has to be severe enough to produce a detectable defect in sleep-like behavior. Loss-of-function also results in lethality if it is too severe. Thus, *egl-30*(*n715sd*) may represent a rare allele that is strong enough to cause sleep-like state defects, yet weak enough not to cause lethality. Because we do not know the exact nature of *egl-30*(*n715sd*), care should be taken when interpreting results obtained with this allele.

How does *egl-30* relate to other genes that have been implicated in the control of sleep-like behavior in *C. elegans*? The *egl-30* pathway is well understood in *C. elegans* and additional factors of this pathway are known. Previous work suggested that Galphaq acts with Galphas and Galphao to control locomotion. Galphas acts through cAMP produced by an adenylate cyclase that is encoded by *acy-1* and gain-of-function mutant that have increased cAMP signaling have been described [23,51]. cAMP signaling has been implicated in the control of sleep-like behavior in *C. elegans*: While *acy-1gf* mutants still show quiescence, this quiescence is interrupted by periods of activity [2,6]. We have measured sleep-like behavior in *acy-1gf* during L1 lethargus and found that nose activity during sleep-like behavior was higher than in wild type, consistent with previous reports. However, nose speed was strongly reduced during sleep-like behavior compared with wake-like behavior (Figure 1B). Thus, the phenotype of *acy-1gf* differs from both *egl-30gf* and *egl-30sd*, suggesting that Galphas and Galphaq signaling have different roles in sleep-like state regulation. Galphao is encoded by *goa-1*. Previous studies showed that *goa-1* negatively regulates locomotion by inhibiting *egl-30*. Thus, *goa-1* mutants have a hyperactive *egl-30* pathway [31,52]. This could explain why the *goa-1* null mutant has a similar sleep-like state phenotype as the *egl-30* gain-of-function mutation. We only assayed the locomotion phenotype of a *goa-1* mutant during lethargus. Previous work assayed response latency to the aversive stimulant octanol and concluded that a *goa-1* null mutant has reduced responsiveness to octanol during lethargus [2]. A *goa-1* gain-of-function mutation, however, has more sleep-like behavior (Figure 1B). The *goa-1* sleep-like phenotypes thus are not the opposite of the *egl-30* phenotypes, suggesting that Galphao and Galphaq have overlapping yet distinct roles in sleep-like state regulation. Our initial analysis also included a hypomorphic allele of *ric-8*, which encodes the guanine nucleotide exchange factor for *egl-30* [23,26]. *ric-8* mutants were almost as hypoactive as *egl-30sd* mutants and also had less relative reduction of activity during sleep-like behavior, consistent with a role of *egl-30* in sleep-like state regulation. EGL-30 has been shown to activate two downstream effectors, the phospholipase C, EGL-8, and the Rho-GEF domain of TRIO/UNC-73 [14,31]. However, we could not find a sleep-like phenotype in neither *egl-8* nor *unc-73* mutants (Figure 1B). The pathway that controls sleep-like behavior downstream of *egl-30* thus remains unknown. *egl-30* may require both pathways redundantly, or may signal through a yet unknown pathway. We could not test worms with mutations in both *egl-8* and *unc-73*, because this condition is embryonic lethal [31].

Is there any relationship between genetic control of *C. elegans* sleep-like behavior and sleep-like states in other animals? We do not know the answer yet. But it is interesting that Galphaq is highly conserved and serves a role not only in *C. elegans* neurons but also in vertebrate brains. In mammals, thalamocortical relay neurons display different modes of firing [53,54], thought to control signal transmission during sleep and wake and arousal [55]. Firing mode change can be achieved by a small shift in membrane potential [53,54]. In one report using brain slices, this small shift has been suggested to be controlled by Galphaq [56]. In *C. elegans* we also see a small shift in basal neural activity [4] and an involvement of Galphaq. Future work should reveal the relationship of these different systems.

## Supporting Information

Figure S1 **Statistical Analysis of nose speed, immobility and reduction of nose speed in *egl-* 30 mutants, *egl-30* rescue mutants and hypoactive mutants during wake-like and sleep-like behavior.** Mean velocity of nose speeds in wild type, *egl-30* mutants, *egl-30* rescue mutants and hypoactive mutants during A wake-like state and B sleep-like state.  Percentage of immobility < 0.5µm/s in wild type, *egl-30* mutants, *egl-30* rescue mutants and hypoactive mutants during C wake-like state and D sleep-like state.  E Percentage of nose speed reduction during lethargus in wild type, *egl-30* mutants, *egl-30* rescue mutants and hypoactive mutants. Errors are SEM. Statistical test is two- sample t-test. * denotes statistical significance at P<0.05, ** denotes statistical significance at P<0.01, *** denotes statistical significance at P<0.001.
(TIF)
