# Supplementary figures and images for: Reduced Sleep-Like Quiescence in Both Hyperactive and Hypoactive Mutants of the Galphaq Gene egl-30 during lethargus in Caenorhabditis elegans
Source: PLoS One. 2013 Sep 20;8(9):e75853. doi: 10.1371/journal.pone.0075853 (PMC3779211; doi:10.1371/journal.pone.0075853)

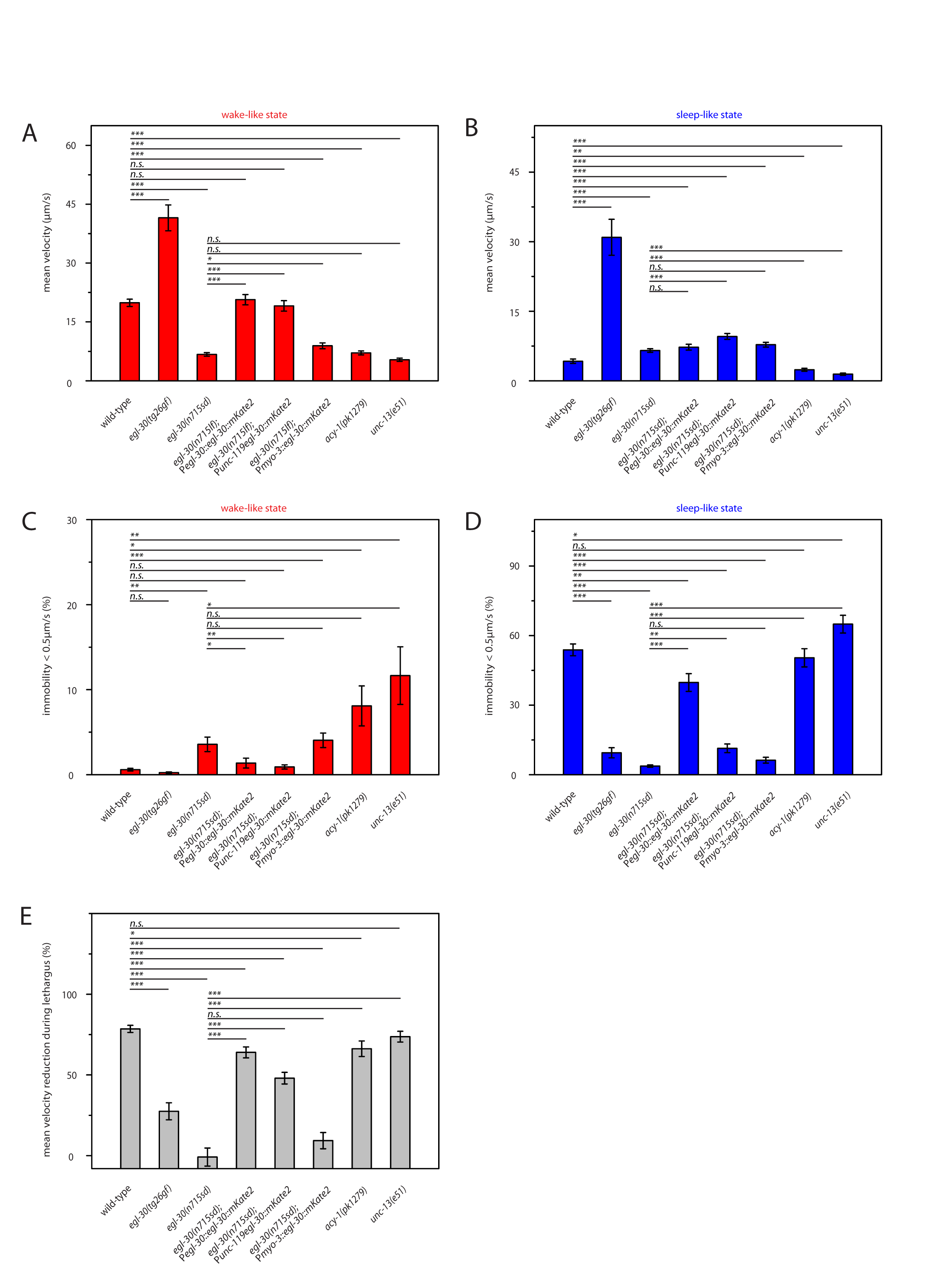

Supplement: Figure S1 — Statistical Analysis of nose speed, immobility and reduction of nose speed in egl- 30 mutants, egl-30 rescue mutants and hypoactive mutants during wake-like and sleep-like behavior. Mean velocity of nose speeds in wild type, egl-30 mutants, egl-30 rescue mutants and hypoactive mutants during A wake-like state and B sleep-like state. Percentage of immobility < 0.5µm/s in wild type, egl-30 mutants, egl-30 rescue mutants and hypoactive mutants during C wake-like state and D sleep-like state. E Percentage of nose speed reduction during lethargus in wild type, egl-30 mutants, egl-30 rescue mutants and hypoactive mutants. Errors are SEM. Statistical test is two- sample t-test. * denotes statistical significance at P<0.05, ** denotes statistical significance at P<0.01, *** denotes statistical significance at P<0.001. (TIF) [file pone.0075853.s001.tif]
